# A comparison of men and women undergoing septoplasty—the Swedish national septoplasty register

**DOI:** 10.3389/fsurg.2023.1223607

**Published:** 2023-07-31

**Authors:** Lars Pedersen, Kenneth Holmberg, Cecilia Ahlström Emanuelsson, Linus Schiöler, Sverre Steinsvåg, Johan Hellgren

**Affiliations:** ^1^ Institute of Clinical Sciences, University of Gothenburg, Gothenburg, Sweden; ^2^ Department of Otorhinolaryngology, Head & Neck Surgery, Skåne University Hospital, Lund, Sweden; ^3^ Lund University, Lund, Sweden; ^4^ Department of Occupational and Environmental Medicine, Sahlgrenska University Hospital, Gothenburg, Sweden; ^5^ Institute of Medicine, University of Gothenburg, Gothenburg, Sweden; ^6^ Department of Otorhinolaryngology, Head & Neck Surgery, Sørlandet Hospital, Kristiansand, Norway; ^7^ University of Bergen, Bergen, Norway; ^8^ Department of Otorhinolaryngology, Head & Neck Surgery, Sahlgrenska University Hospital, Gothenburg, Sweden

**Keywords:** cross-sectional register study, sex, nasal obstruction, prom, septoplasty, turbinoplasty

## Abstract

**Objective:**

Men represent more than two-thirds of septoplasty patients in many studies, but differences between men and women in terms of patient selection or outcome are seldom reported. This study aims to investigate whether women undergoing septoplasty differ from men in critical variables before and after surgery, in a large national sample of septoplasties.

**Design:**

Cross-sectional register study.

**Participants:**

The study includes 2,532 patients from the National Swedish Septoplasty Register undergoing septoplasty with or without additional turbinoplasty on the indication of nasal obstruction in 2014–2019. Patients in the register have not been preselected.

**Main outcome measures:**

Preoperative variables and postoperative outcome were compared between men and women.

**Results:**

Men accounted for 1,829 (72%) of the patients. There was no significant difference between men and women in severity of self-reported nasal obstruction or type of surgery performed (septoplasty with or without turbinoplasty). Mean postoperative nasal obstruction 12 months after surgery and overall satisfaction with the result were similar. Women, however, reported more complications 12 months postoperatively, while men reported more problems with snoring and obstructive sleep apnea preoperatively.

**Conclusion:**

In this large national patient cohort undergoing septoplasty, we found no differences in preoperative nasal obstruction or postoperative patient-rated outcome in men and women undergoing septoplasty, despite the fact that 72% of the patients were men. It thus remains unclear why women are under-represented in septoplasty surgery in this and many other cohorts.

## Introduction

Septoplasty is the recommended surgical intervention to relieve structural nasal obstruction due to a deviated nasal septum. The efficacy of this operation is well known from many studies using objective measurements of nasal airflow and resistance, but this has only recently been confirmed in a first randomised controlled trial compared with non-surgical treatment (1, 2). Regarding patient reported outcome measures (PROMS), including subjective perception of nasal obstruction, the results of improvement 6–12 months after surgery vary around 70%–80% (3, 4).

Septal deviations are common in the adult population. In one study of 2,589 adult unselected patients attending 17 ENT departments in 14 countries for various ENT complaints and examined with anterior rhinoscopy, 89.2% exhibited septal deviations, with only small differences between genders (5). A straight septum was found in in 15.4% of the females and 7.5% of the males. Although septal deviations are common in the population, only a minority of all septal deviations cause symptoms that result in a septoplasty. Given the relatively small differences in the natural distribution of septal deviations between men and women, it is surprising that several recent studies analysing predictors and outcome in septoplasty report a considerable male predominance in the septoplasty cohorts (4). Data from the Swedish National Septoplasty Register (SNSR) show that around 70% of the patients are males, which is in line with other studies (1, 4, 6, 7). It has been proposed that men are more involved in nasal traumas related to sports activities, assaults and motor vehicle accidents and therefore experience more problems with their nasal breathing due to a nasal obstruction (8).

There are studies that report a more equal distribution of women and men undergoing septoplasty in unselected cohorts. One study from Italy included 494 patients, of which 284 were women (57%). It found that gender was not an important predictor of the postoperative result after septoplasty with turbinoplasty after 6 months (9). Another study, including 54% women, concluded that gender was not associated with the baseline Nasal Obstruction and Septoplasty Effectiveness Scale (NOSE) score (10).

The SNSR contains a large national Swedish cohort of patients operated with septoplasty with and without turbinoplasty. In a previous study from the SNSR, in which 76% of the patients were men, we found that women had a significantly higher odds ratio when it came to reporting a better postoperative result *six months* after surgery than men in 5,865 patients undergoing surgery from 2003 to 2012 (4). In 2013, the SNSR was extended to include more specific questions on nasal symptoms before and *twelve months* after surgery. In the present study the aim was to compare male and female patients undergoing septoplasty with or without turbinoplasty due to nasal obstruction in 2014–2019 to identify any differences in patient selection and outcome related to sex. We also wanted to investigate whether the results of the previous study were consistent in the more extensive data from the updated register.

## Material and methods

### Participants

The SNSR is operated by the Swedish Association for Otorhinolaryngology, Head & Neck Surgery, in association with the Swedish Association of Local Authorities and Regions (SKR). Patients undergoing septoplasty answer a preoperative questionnaire on nasal symptoms. The operating surgeon also reports on the type of surgery and indication for surgery. Twelve months after surgery, the patient answers a questionnaire on postoperative nasal symptoms and complications, if applicable. The SNSR has been described in more detail elsewhere (11).

### Inclusion criteria

**Figure 1:**
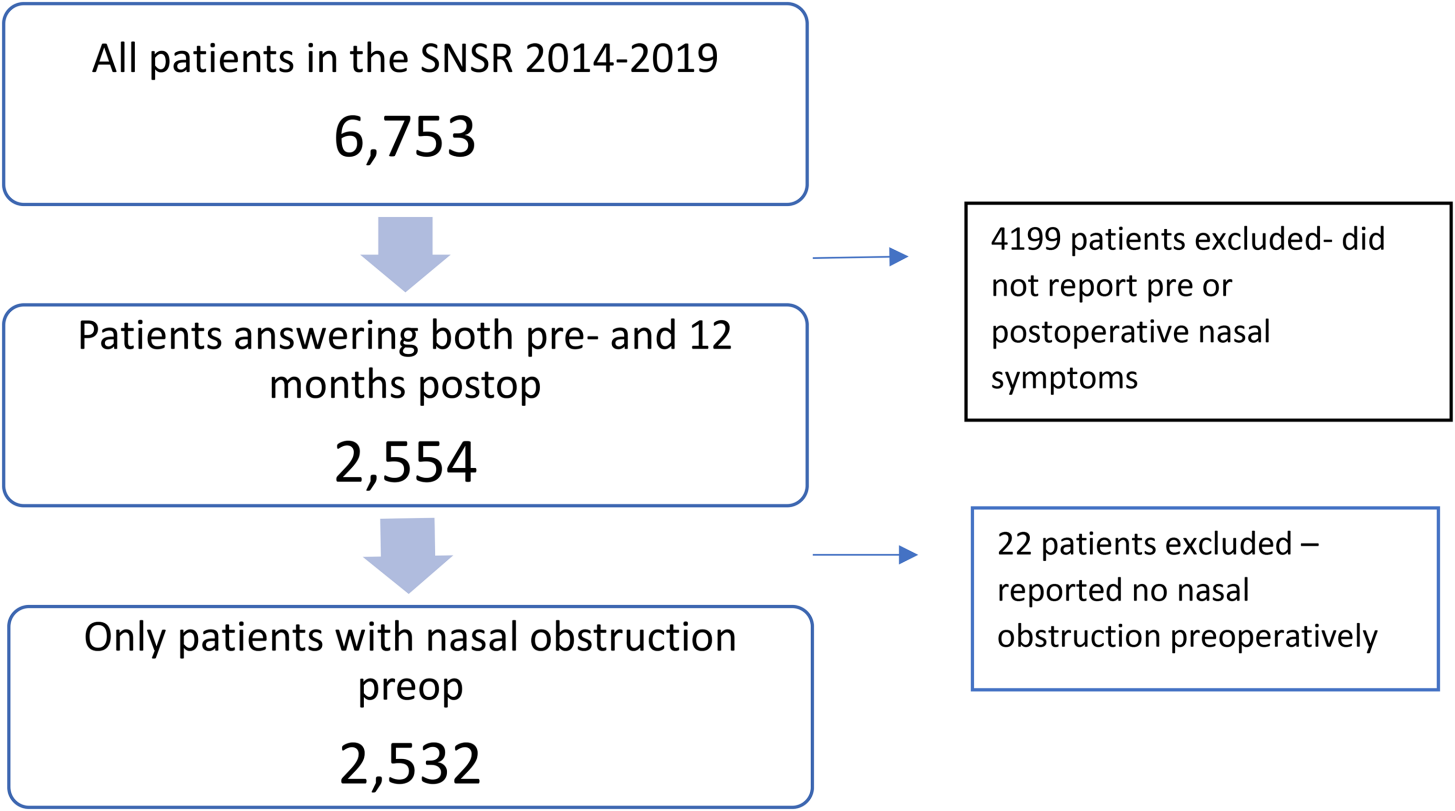
Flow diagram of the study population. Patients not answering both the pre- and postoperative questionnaire after 12 months (*n* = 4,199) and patients reporting “no nasal obstruction” preoperatively were excluded (*n* = 22). After exclusion, *N* = 2,532.

The present study consists of 2,532 patients from Sweden undergoing surgery from 2014 to 2019. The flow chart for patient selection is shown in Figure 1. Only patients who answered both the preoperative and the 12-month postoperative questionnaire were included in this study. A few patients who reported “no nasal obstruction” preoperatively were also excluded. Table 1 shows the baseline data of the study population, stratified by gender. The data have been extracted from the preoperative questionnaire.

**Table 1:** Baseline data on the study population from the SNSR in 2014–2020, *N* = 2,532.

	Men	Women	Difference	All	Missing
Sex, *n* (%)	1,829 (72.2)	703 (27.8)		2,532	0
Median BMI, kg/m^2^ (IQR)	25.6 (23.6–28.4)	24.4 (22.0–27.8)	1.2 (0.7–1.7)^b^	25.3 (23.1–28.4)	209
Median age at surgery, years (IQR)	35 (26–48)	35 (25–49)	0 (−1.9;1.9)^b^	35 (26–48)	0
Smoking habits *n* (%)			*p* = 0.36^c^		189
Non-smokers	1,319 (77.9)	518 (80)	−1.8 (−5.5;1.9)^d^	1,837 (78.4)	
Smoking sometimes	159 (9.4)	49 (7.5)		208 (8.9)	
Smoking daily	215 (12.7)	83 (12.8)		298 (12.7)	
Nasal polyps, *n* (%)	49 (3.1)	16 (2.6)	0.5 (−1.0;2.0)	65 (3.0)	340
Rhinitis, *n* (%)	34 (21.1)	145 (23.5)	−2.5 (−6.4;1.4)	479 (21.8)	330
Snoring, *n* (%)	566 (36.2)	124 (20.4)	15.8 (11.8;19.8)	690 (31.8)	359
OSAS, *n* (%)	201 (12.8)	22 (3.6)	9.2 (6.9;11.4)	223 (10.2)	346
Pathological rhinomanometry^a^			*p* = 0.32		0
Yes	1,033 (56.5)	382 (54.3)	0.9 (−2.7;4.4)^e^	1,415 (55.9)	
No	144 (7.9)	49 (7.0)		193 (7.6)	
No investigation	652 (35.6)	272 (38.7)		924 (36.5)	
Time of day problem			*p* = 0.03^c^		45
Day	83 (4.6)	26 (3.8)		109 (4.4)	
Night	315 (17.5)	93 (13.5)		408 (16.4)	
Both	1,402 (77.9)	568 (82.7)	−4.8(−8.2;−1.4)^f^	1,970 (79.2)	

^a^ This is filled out by the ENT surgeon in the preoperative questionnaire.

^b^ Difference between medians and 95% CI calculated by quantile regression.

^c^ Overall Pearson's chi-squared *p*-value.

^d^ No smoking vs. Any smoking.

^e^ Among those with investigation.

^f^ Both vs. day or night.

### The questionnaires

The SNSR is based on four questionnaires filled out, at the preoperative visit, at the operating theatre, 1 month postoperatively and 12 months postoperatively.

Symptoms of nasal obstruction are reported by the patients before and twelve months after surgery and graded as *none, mild, moderate, severe*, side of nasal obstruction, day- and/or night-time symptoms and activity impairment (none, mild, moderate, severe) due to the nasal obstruction.

The question used to assess activity limitation due to nasal obstruction (for example, work, studies, leisure activities) and sleep has been adapted from the “Allergic rhinitis and its impact on asthma” (ARIA) consensus document. This item was graded as *none, mild, moderate or severe*.

The ENT surgeon who diagnosed the patient reported on side of the septal deviation (right, left, both), co-morbidities, if rhinomanometry was used and if it was deemed pathological by the examining ENT surgeon and which surgical procedure that was planned.

The type of surgery performed (septoplasty with or without turbinoplasty) was reported by the ENT surgeon performing the septoplasty.

Postoperative complications were assessed with a questionnaire mailed or e-mailed to the patient *one month* after surgery. The patient was asked about any unplanned visits to healthcare due to postoperative complications (i.e., bleeding, pain, infection or other) within *the first month* after surgery.

A final questionnaire was mailed or e-mailed to the patient 12 months after surgery. Patients were asked to report their perceived level of nasal obstruction, the impact the obstruction had on daily activities and sleep and the side of the nasal obstruction. They were also asked whether the result of the operation came up to their expectations and whether they had suffered any unexpected adverse effects 12 months after surgery. Patients who rated their nasal obstruction as one level better 12 months after septoplasty compared with preoperatively were defined as “improved”.

### Ethical considerations

Patients received information about the register at the clinical visits and in the accompanying written information sent out with the postoperative questionnaire. The return of a completed questionnaire was regarded as informed consent. The questionnaires did not pose any hazard to the participants. The study was approved by The Swedish Ethical Review Authority, Dnr 2021-01559.

## Statistical analysis

Descriptive statistics, using means medians and standard deviations interquartile range for continuous variables and frequencies and percentages for categorical variables, were used to summarise the characteristics of the study population from questionnaire responses. For dichotomous variables the difference between proportions were calculated as the difference in percentage points with 95% confidence intervals (CI:s) calculated using the Wald method. For polytomous variables some CI:s were calculated by either collapsing two categories or removing one category. In these cases, an overall *p*-value Comparisons between groups were made were calculated using Pearson's chi-square test. *p*-Values calculated using the Cochran-Armitage test were presented in addition to the chi-squared test in cases where both null hypotheses were of interest. For continuous variables, the difference between medians were calculated by quantile regression with 95% CI:s calculated using the Markov chain marginal bootstrap method. Odds ratios were calculated by ordinal logistic regression with a cumulative logit link to determine the association between patient-rated symptoms and different predictors. Statistical calculations were made using SAS 9.4M6 (SAS Institute, Cary, NC). All test were two-sided, *p*-values of <0.05 were considered statistically significant and 95% confidence level were used for all CI:s.

## Results

A total of 2,532 patients were included in the study, Figure 1. The baseline data of the included patients can be seen in Table 1. Of the patients, 1,829 were men (72%) and 703 (28%) women. There was a similar distribution between men and women regarding mean age, tobacco smoking, rhinitis, presence of nasal polyps and presence of preoperative “pathological” rhinomanometry. Men reported more problems with snoring and sleep apnea. Men reported more symptoms at night.

In the comparison between men and women, self-reported nasal obstruction before septoplasty with or without turbinoplasty was similar as well as postoperative nasal obstruction 12 months after surgery Table 2. Overall satisfaction with the surgical result after twelve months was similar, and so was the type of surgery performed (septoplasty with or without additional turbinate surgery).

**Table 2:** Self-reported nasal obstruction preoperatively and postoperatively (graded no, mild, moderate, severe) in 2,532 patients undergoing septoplasty with or without turbinoplasty from the SNSR 2014–2019, stratified by gender.

	Nasal obstruction			
	Preop^a^		Postop^b^	
*n* (%)	Female	Male	Female	Male
No			168 (23.9)	428 (23.4)
Mild	85 (12.1)	235 (12.8)	257 (36.6)	667 (36.5)
Moderate	334 (47.5)	832 (45.5)	184 (26.2)	490 (26.8)
Severe	284 (40.4)	762 (41.7)	94 (13.4)	244 (13.3)

^a^*p* chi-square = 0.64, *p* trend = 0.87.

^b^*p* chi-square = 0.99, *p* trend = 0.81.

Women reported more complications 12 months postoperatively than men. Just over 27% of the women reported some kind of complication, compared with 23% of the men. The most common complications reported were change in smell and change in the shape of the nose.

**Figure 2:**
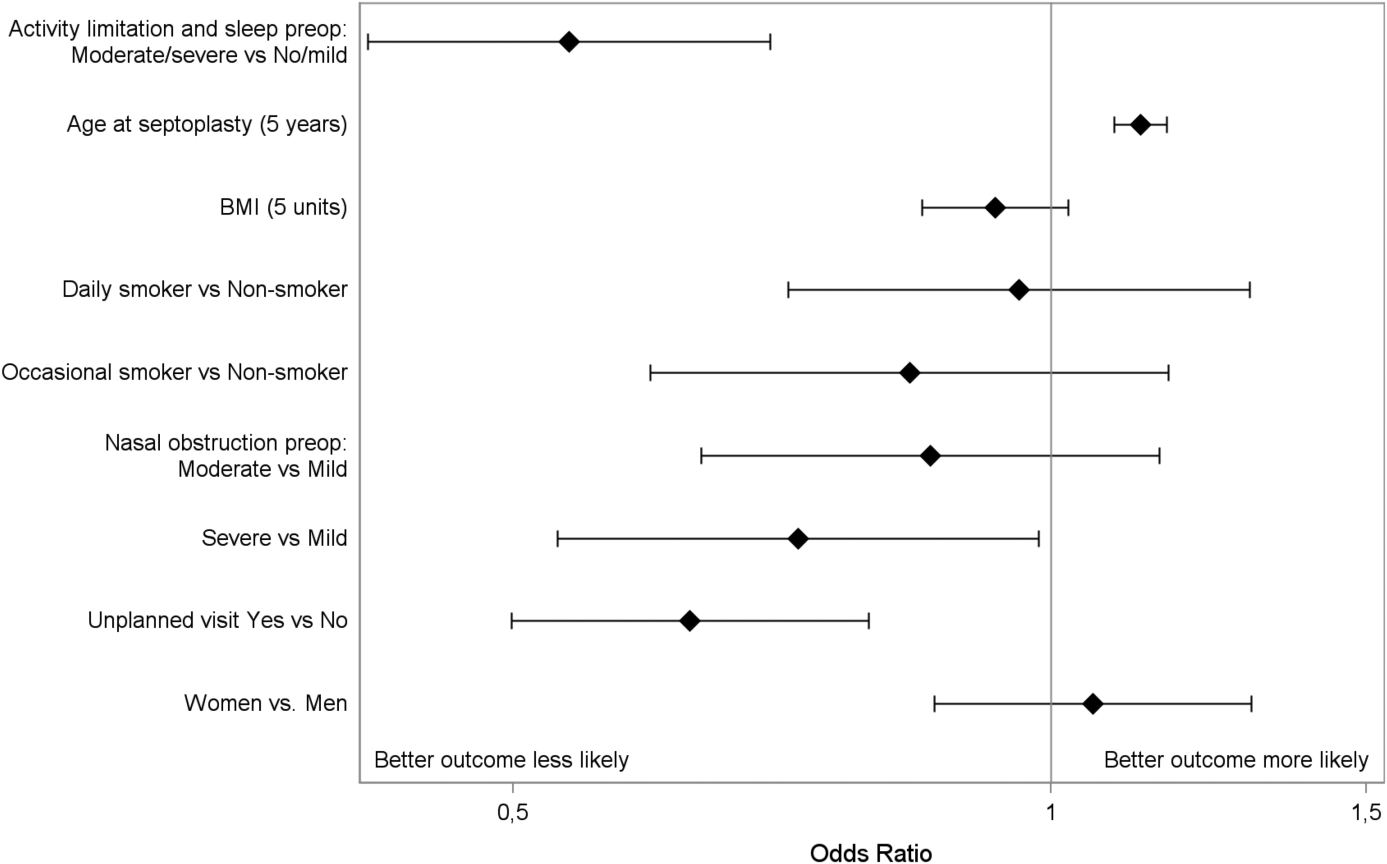
Multivariable ordinal logistic regression of predictors in relation to outcome after septoplasty. Presented as odds ratios with 95% confidence limits and including unplanned visits to the hospital within 1 month after surgery, moderate or severe activity limitation preoperatively due to nasal obstruction, smoking (occasional, daily), BMI (+5 units), degree of nasal obstruction (mild vs. moderate, mild vs. severe), gender and age (+5 years).

Odds ratios for a better outcome after surgery was calculated in a multivariable ordinal logistic regression model. No reported, unplanned visits postoperatively were related to a better outcome 12 months after surgery. This sentence should be altered: Higher age also predicted a better outcome, while having a moderate to severe effect on daily activity preoperatively did not. There were no differences between men and women, Figure 2.

**Figure 3:**
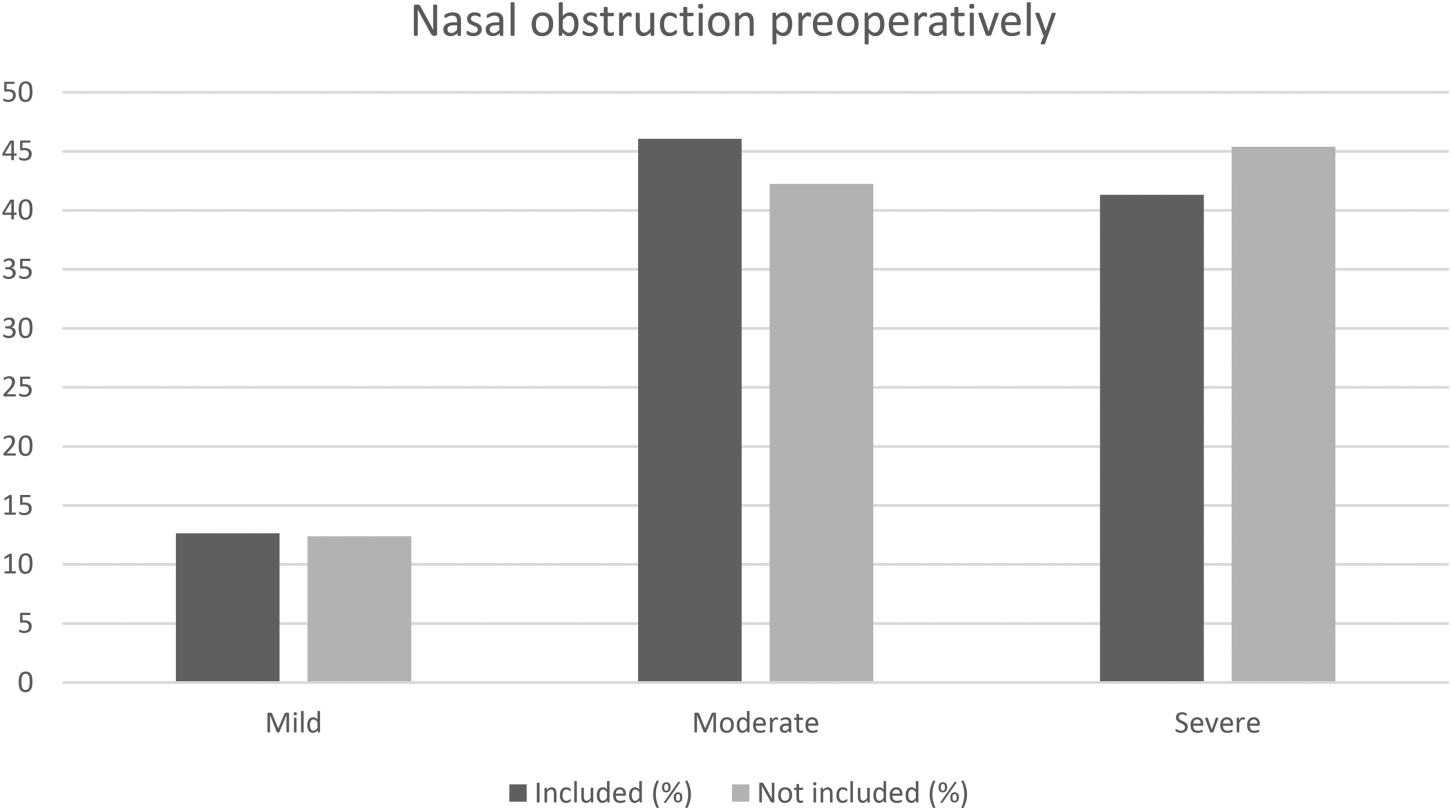
Comparison of nasal obstruction preoperatively between the patients included in the study (answering both the preoperative and postoperative questionnaire, *N* = 2,532) and the patients not included (not answering the postoperative questionnaire, *N* = 2,534). *p* Chi-square = 0.01, *p* trend = 0.02.

**Figure 4:**
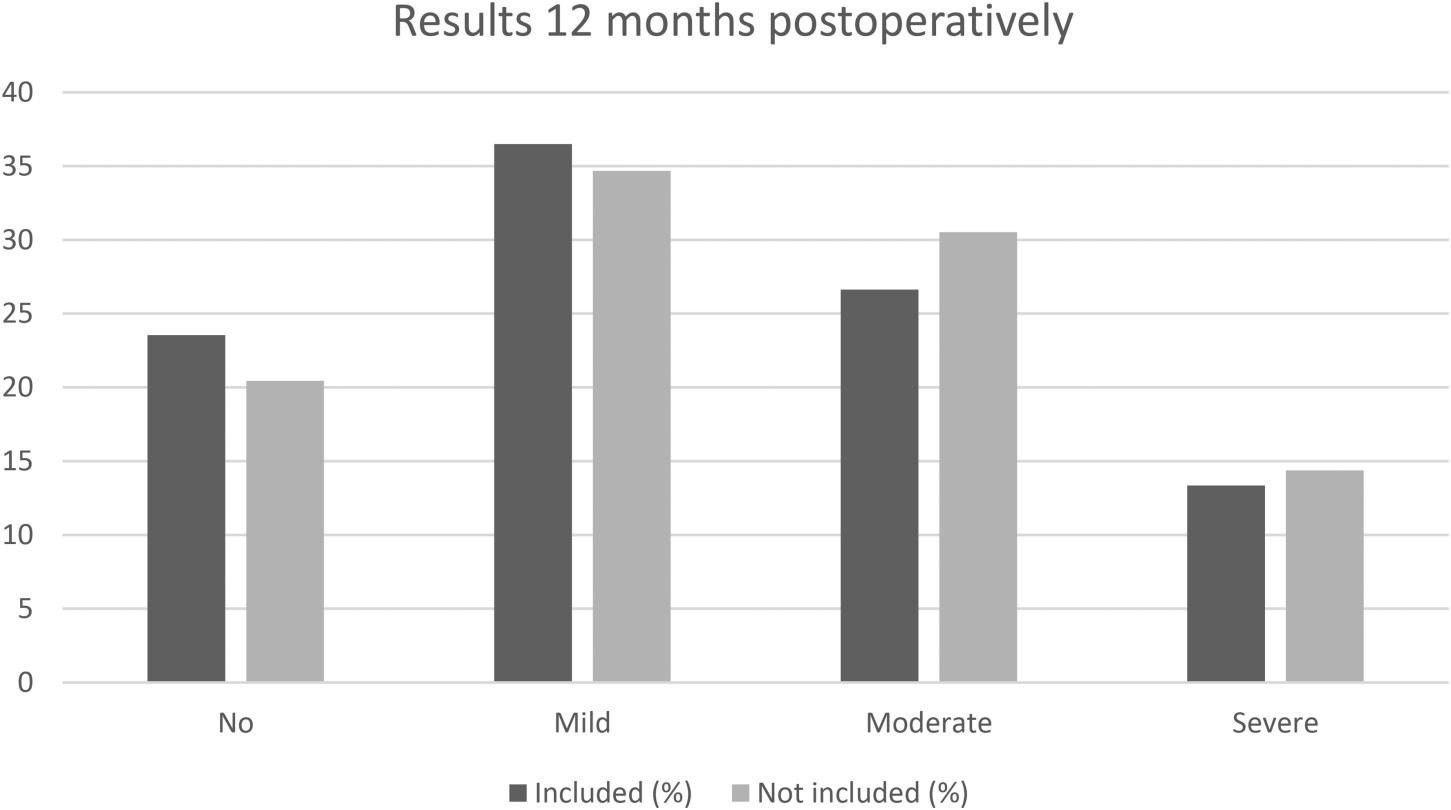
Comparison of nasal obstruction after 12 months between the patients included in the study (answering both the preoperative and postoperative questionnaire, *N* = 2,532) and the patients not included (not answering the preoperative questionnaire, *N* = 675). *p* Chi-square = 0.11, *p* trend = 0.03.

We analysed the patients who were excluded from this study because they did not answer both the pre- and postoperative questionnaire regarding nasal symptoms. We found that the excluded patients lacking postoperative data, reported slightly worse nasal obstruction before surgery, and the excluded patients who were lacking preoperative data, reported slightly worse nasal obstruction twelve months after surgery. The 2,423 patients answering only the preoperative questionnaire are shown in Figure 3, while the 675 patients answering only the postoperative questionnaire are shown in Figure 4.

## Discussion

### Synopsis of key/new findings

In this large national study of patients undergoing septoplasty with or without turbinoplasty in which 72% were men, we found no difference between men and women in terms of mean age at surgery, pre- and postoperative self-reported nasal symptoms, the frequency of additional turbinoplasty, or overall satisfaction twelve months after surgery. The reason why women are underrepresented in septoplasty in many studies including this large sample remains unclear, but we found no indication of selection bias.

### Comparison with other studies

As seen in many studies of septoplasty cohorts from different parts of the world, this large national cohort exhibited a male domination of 72% (4, 7, 12). The women, comprising 703 patients, showed no difference compared with the men regarding nasal obstruction before surgery or nasal obstruction twelve months after surgery or in overall satisfaction with surgery. This is in agreement with the postoperative results from previous studies, where no difference between genders was reported (12, 13). We have reported a higher postoperative satisfaction rate *six months* after septoplasty in women undergoing septoplasty. This was in a cohort of 5,865 septoplasty patients from the SNSR with a mean age of 36 years undergoing surgery in 2003–2012, in which 76% were men. At the time, the SNSR focused only on patient satisfaction after surgery and did not include questions on specific nasal symptoms, such as the grading of nasal obstruction before and after surgery. A direct comparison with the present data is therefore not possible. The follow-up time was also shorter (6 months vs. 12 months) which could have affected the result.

The present results, being in line with that from several other studies, however, indicate that there is no clinically relevant selection bias related to sex between men and women undergoing septoplasty.

In a large retrospective study including almost 3,000 patients analysing the epidemiological characteristics of nasal bone fractures, men were clearly overrepresented. The leading cause was traffic accidents, followed by violent assault (14). This supports the idea that men are more exposed to nasal trauma that may cause nasal obstruction to a greater extent in men than in women. Other similar studies of facial fractures support this finding (15). It is therefore interesting to see that the mean age at surgery in this study was the same for both men and women. If more trauma was the cause of the male predominance in septoplasty, it would likely have skewed the men to surgery at an earlier age in life, but that was not the case in our material, where the median age for surgery was 35 years in both men and women.

Smoking is known to have a negative impact on the nasal mucosa and is a risk factor for nasal obstruction and chronic rhinosinusitis (CRS) (16). Historically, smoking has been more prevalent in men, but during the last few decades this difference has levelled out in Sweden. Our data also confirm that smoking was not more common in men than in women undergoing septoplasty in this study and is therefore an unlikely contributory factor to the predominance of men undergoing surgery.

In the SNSR, almost 50% of all the septoplasties reported were combined with a turbinoplasty. Turbinoplasty is typically added to the septoplasty if the repositioning of the septum leads to obstruction on the previously patent side of the nose due to turbinate hypertrophy. Another common issue is to address concomitant inflammatory mucosal disease such as allergy. No difference in allergic rhinitis prevalence was, however, seen in this study between men and women and no difference in the frequency of an ad on turbinoplasty procedure.

In the literature, gender differences relating to respiratory function show that respiratory disturbances, including sleep apnea syndrome, are less common in women than men up to menopause (17). As expected, men report more problems with snoring and sleep apnea in our study with a median age of 35 (Table 1). Snoring in men could be a social problem and lead to the diagnosis of a deviated septum and a septoplasty to a greater extent. Another possible effect of hormonal influence has been studied by Macsali et al. who examined the menstrual cycle and respiratory symptoms in almost 4,000 women. They found that the symptoms varied significantly during the menstrual cycle (18). Hormonal differences could thus also play a role in the nasal airway and “protect” women until their menopause around 50 years of age. This could be one explanation for the relatively small number of women undergoing septoplasty compared with men.

There are theories that indicate that men have an increased perception of “sickness” that could be related to the immunosuppressive role of testosterone (19, 20). If the predominance of men in septoplasty studies reflects men suffering more from their respiratory problems, including nasal breathing, they might be more likely than women to seek healthcare and have their septal deviation diagnosed and treated.

Since a septal deviation is common in the population, factors other than the structural obstruction as such could affect the sensation of a blocked nose. Malik et al. suggest that an impaired ability to lateralise menthol and the cooling sensation rather than a change in airflow in the presence of a deviated septum could drive the symptoms of obstruction (21). Other studies also support this theory of impaired trigeminal function as a cause of nasal obstruction, but no differences between men and women have been observed (22).

### Clinical applicability of the study

In the whole study population the odds ratios of a better outcome after surgery are in line with the results in our previous study from the SNSR (4). Unplanned extra visits postoperatively due to postoperative bleeding, infection or pain were related to a poorer outcome. We believe this could indicate complications after the surgery that may have resulted in removal of packaging or infection affecting the healing process. Unplanned events could also have had a negative impact on the patient's perception of the success of the surgical procedure. Moreover, younger age predicts a poorer outcome. We have previously found that higher age predicts a better outcome after septoplasty in two studies from the SNSR. Differences in the perception of symptoms with older age and the role of increased life experience of stressful events, could affect the personal experience in younger vs. older patients. Healing conditions can also differ with age and higher age has been associated with having larger nasal cavities, measured with acoustic rhinometry.

Reporting activity limitations preoperatively made it less likely for a better outcome (reported nasal obstruction) 12 months after surgery. Hence not be confused with predicting an improvement after surgery as such. No significant differences between men and women were seen in the analyses. We have previously reported that in patients with severe nasal obstruction preoperatively, 81% had improved 12 months after surgery and in patients with moderate nasal obstruction before surgery, the improvement rate dropped to 57%.

### Weaknesses

This study has limitations. The data in the SNSR is related to PROMS. There were no objective data of nasal air-flow resistance or intranasal geometry, such as rhinomanometry or acoustic rhinometry available in this study. PROMS and objective measures, however, often show conflicting results (1, 4). It is thus important to consider both PROMS and objective measures in the follow up of septoplasty surgery as partly different but complementary outcome measures. It is a very large national multi center cohort of 2,532 patients undergoing septoplasty, however, the follow-up time of 12 months does not indicate the results in the long term for these patients. We have no access to data from the objective methods used, such as rhinomanometry, to verify the effect of the surgery on nasal airflow and resistance. Patients in this study were also excluded because they did not answer one of the questionnaires and the reporting to the register of all septoplasties in Sweden varies between different years (42%–60%).

## Conclusion

In this large national patient cohort undergoing septoplasty, we found no differences in preoperative nasal obstruction or postoperative patient-rated outcome in men and women undergoing septoplasty, despite the fact that 72% of the patients were men. It thus remains unclear why women are under-represented in septoplasty surgery in this and many other cohorts.

## Data Availability

The original contributions presented in the study can be found at: https://sep.registercentrum.se/, further inquiries can be directed to the corresponding author.
