# Supplementary material for: A comparison of men and women undergoing septoplasty—the Swedish national septoplasty register
Source: Front Surg. 2023 Jul 31;10:1223607. doi: 10.3389/fsurg.2023.1223607 (PMC10423992; doi:10.3389/fsurg.2023.1223607)
Supplement: Supplementary file 1 [file Table1.docx]

Supplementary Material

A comparison of men and women undergoing septoplasty – The Swedish National Septoplasty Register

**Lars Pedersen, Kenneth Holmberg, Cecilia Ahlström Emanuelsson, Linus Schiöler, Sverre Steinsvåg,** **Johan Hellgren Correspondence:** Johan Hellgren: johan.hellgren@gu.se

# Supplementary Figures and Tables

**Supplementary Table 1.** Estimates and standard errors from Multivariable ordinal logistic regression. Cumulative logit proportional odds model, i.e. logit(Pr(Y≤i|**x**), i=1,2,3, where 1, 2 and 3 denotes No, Mild and Moderate nasal obstruction 12 months after surgerery respectively.

| **Parameter** | **Response** | **Estimate** | **Standard Error** |
| --- | --- | --- | --- |
| **Intercept** | **No** | -1.6034 | 0.2706 |
| **Intercept** | **Mild** | 0.1060 | 0.2669 |
| **Intercept** | **Moderate** | 1.6864 | 0.2729 |
| **Age5years** |  | 0.1149 | 0.0173 |
| **Gender** | **Female** | 0.0266 | 0.0521 |
| **ObstructionPreop** | **Moderate** | 0.00490 | 0.0678 |
| **ObstructionPreop** | **Severe** | -0.1656 | 0.0733 |
| **BMI5units** |  | -0.0722 | 0.0481 |
| **SmokingHabits** | **Smoking daily** | 0.0330 | 0.1105 |
| **SmokingHabits** | **Smoking sometimes** | -0.1078 | 0.1193 |
| **ActivityLimitation** | **Moderate/severe** | -0.3104 | 0.0660 |
| **UnplannedVisit** | **Yes** | -0.2327 | 0.0587 |

**Supplementary Table 2.** Estimated covariance matrix for Multivariable ordinal logistic regression.

| Parameter | Intercept No | Intercept Mild | Intercept Moderate | Age 5years | Female | Obstr Moderate | Obstr Severe | BMI 5 units | Smoke daily | Smoke sometimes | Act.Lim. Mod/Sev | Unplan. Visit |
| --- | --- | --- | --- | --- | --- | --- | --- | --- | --- | --- | --- | --- |
| InterceptNo | 0.073231 | 0.070253 | 0.06946 | -0.00133 | 0.000216 | -0.00122 | 0.000373 | -0.01024 | 0.002502 | 0.001636 | -0.00143 | 0.002161 |
| Intercept Mild | 0.070253 | 0.071244 | 0.070036 | -0.0012 | 0.000248 | -0.00122 | 0.000177 | -0.01031 | 0.002536 | 0.001518 | -0.00182 | 0.001898 |
| Intercept Moderate | 0.06946 | 0.070036 | 0.074469 | -0.00111 | 0.000271 | -0.00119 | 0.000039 | -0.01039 | 0.002577 | 0.001405 | -0.00199 | 0.001679 |
| Age 5years | -0.00133 | -0.0012 | -0.00111 | 0.000301 | 0.000011 | -0.00002 | -2E-06 | -0.00021 | -0.00008 | 0.000133 | 0.00004 | -0.00003 |
| Female | 0.000216 | 0.000248 | 0.000271 | 0.000011 | 0.00271 | -0.00007 | 0.000074 | 0.000151 | -0.0002 | 0.000199 | -0.00007 | -0.00019 |
| Obstr Moderate | -0.00122 | -0.00122 | -0.00119 | -0.00002 | -0.00007 | 0.004594 | -0.00029 | 0.000087 | 0.000378 | -0.0001 | -0.00048 | 0.000041 |
| Obstr Severe | 0.000373 | 0.000177 | 0.000039 | -2E-06 | 0.000074 | -0.00029 | 0.00538 | -0.00012 | -0.00016 | -0.00011 | -0.00162 | 0.000131 |
| BMI 5 units | -0.01024 | -0.01031 | -0.01039 | -0.00021 | 0.000151 | 0.000087 | -0.00012 | 0.002317 | -0.00016 | 0.000169 | -0.00016 | 8.85E-06 |
| Smoke daily | 0.002502 | 0.002536 | 0.002577 | -0.00008 | -0.0002 | 0.000378 | -0.00016 | -0.00016 | 0.012211 | -0.01003 | -0.00011 | -0.00021 |
| Smoke sometimes | 0.001636 | 0.001518 | 0.001405 | 0.000133 | 0.000199 | -0.0001 | -0.00011 | 0.000169 | -0.01003 | 0.014228 | 0.000038 | 0.000062 |
| Act.Lim. Mod/Sev | -0.00143 | -0.00182 | -0.00199 | 0.00004 | -0.00007 | -0.00048 | -0.00162 | -0.00016 | -0.00011 | 0.000038 | 0.004352 | 0.000065 |
| Unplan. Visit | 0.002161 | 0.001898 | 0.001679 | -0.00003 | -0.00019 | 0.000041 | 0.000131 | 8.85E-06 | -0.00021 | 0.000062 | 0.000065 | 0.003443 |
