# Supplementary material for: A comparison of men and women undergoing septoplasty—the Swedish national septoplasty register
Source: Front Surg. 2023 Jul 31;10:1223607. doi: 10.3389/fsurg.2023.1223607 (PMC10423992; doi:10.3389/fsurg.2023.1223607)

Figure 1. Flow diagram of the study population. Patients not answering both the pre- and postoperative questionnaire after 12 months (n=4,199) and patients reporting “no nasal obstruction” preoperatively were excluded (n=22). After exclusion, N=2,532.

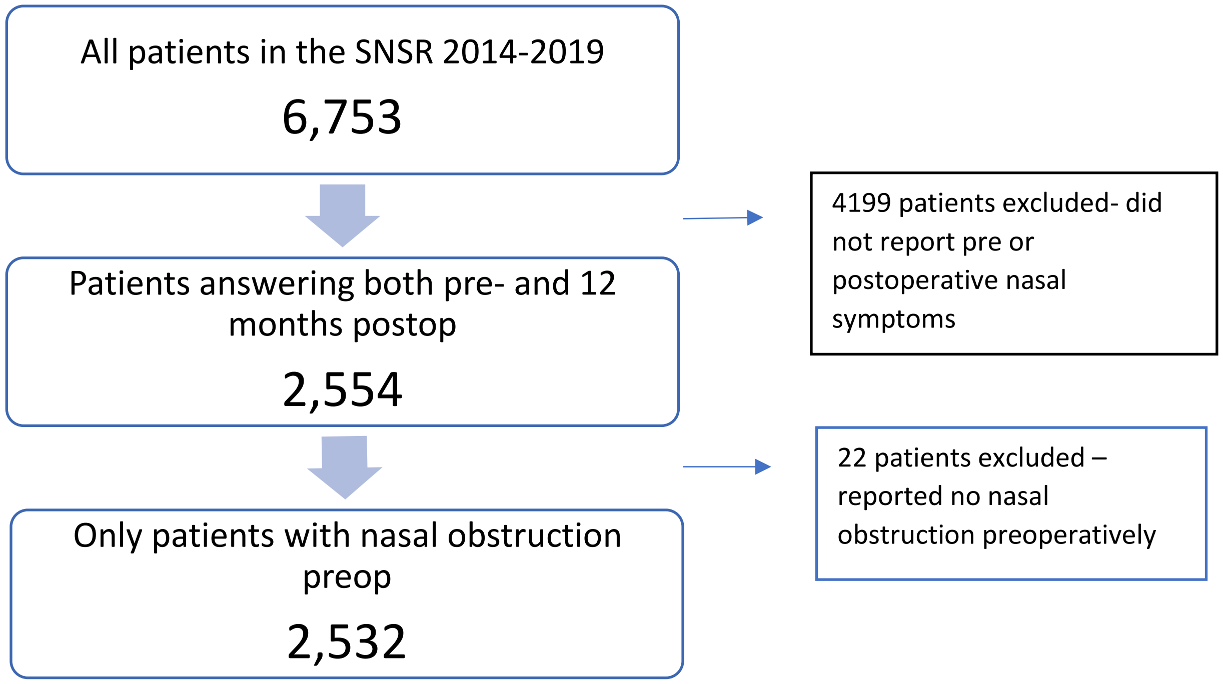

Table 1. Baseline data on the study population from the SNSR in 2014-2020. N=2,532

	men	women	Difference	all	missing
sex, n (%)	1829 (72.2)	703 (27.8)		2532	0
Median BMI, kg/m^2 (IQR)	25.6 (23.6-28.4)	24.4 (22.0-27.8)	1.2 (0.7-1.7)[1]	25.3 (23.1-28.4)	209
Median age at surgery, years (IQR)	35 (26-48)	35 (25-49)	0 (-1.9;1.9)[1]	35 (26-48)	0
			p=0.36[2]		189
smoking habits n (%)					
non-smokers	1319 (77.9)	518 (80)	-1.8 (-5.5;1.9)[3]	1837 (78.4)	
smoking sometimes	159 (9.4)	49 (7.5)		208 (8.9)	
smoking daily	215 (12.7)	83 (12.8)		298 (12.7)	
nasal polyps, n (%)	49 (3.1)	16 (2.6)	0.5 (-1.0;2.0)	65 (3.0)	340
rhinitis, n (%)	34 (21.1)	145 (23.5)	-2.5 (-6.4;1.4)	479 (21.8)	330
snoring, n (%)	566 (36.2)	124 (20.4)	15.8 (11.8;19.8)	690 (31.8)	359
OSAS, n (%)	201 (12.8)	22 (3.6)	9.2 (6.9;11.4)	223 (10.2)	346
pathological rhinomanometry*			p=0.32		0
Yes	1033 (56.5)	382 (54.3)	0.9 (-2.7;4.4)[4]	1415 (55.9)	
No	144 (7.9)	49 (7.0)		193 (7.6)	
No investigation	652 (35.6)	272 (38.7)		924 (36.5)	
time of day problem			p=0.03[2]		45
day	83 (4.6)	26 (3.8)		109 (4.4)	
night	315 (17.5)	93 (13.5)		408 (16.4)	
both	1402 (77.9)	568 (82.7)	-4.8(-8.2;-1.4)[5]	1970 (79.2)	

*This is filled out by the ENT surgeon in the preoperative questionnaire.

[1] Difference between medians and 95 % CI calculated by quantile regression

[2] Overall Pearson’s chi-squared p-value

[3] No smoking vs. Any smoking

[4] Among those with investigation

[5] Both vs. day or night

Table 2. Self-reported nasal obstruction preoperatively and postoperatively (graded no, mild, moderate, severe) in 2,532 patients undergoing septoplasty with or without turbinoplasty from the SNSR 2014-2019, stratified by gender.

	**Nasal obstruction**			
	**Preop[1]**		**Postop[2]**	
n (%)	Female	Male	Female	Male
No			168 (23.9)	428 (23.4)
Mild	85 (12.1)	235 (12.8)	257 (36.6)	667 (36.5)
Moderate	334 (47.5)	832 (45.5)	184 (26.2)	490 (26.8)
Severe	284 (40.4)	762 (41.7)	94 (13.4)	244 (13.3)

[1] p chi-square=0.64, p trend=0.87

[2] p chi-square=0.99, p trend=0.81

Figure 2. Multivariable ordinal logistic regression of predictors in relation to outcome after septoplasty. Presented as odds ratios with 95% confidence limits and including unplanned visits to the hospital within 1 month after surgery, moderate or severe activity limitation preoperatively due to nasal obstruction, smoking (occasional, daily), BMI (+5 units), degree of nasal obstruction (mild vs moderate, mild vs severe), gender and age (+5 years).

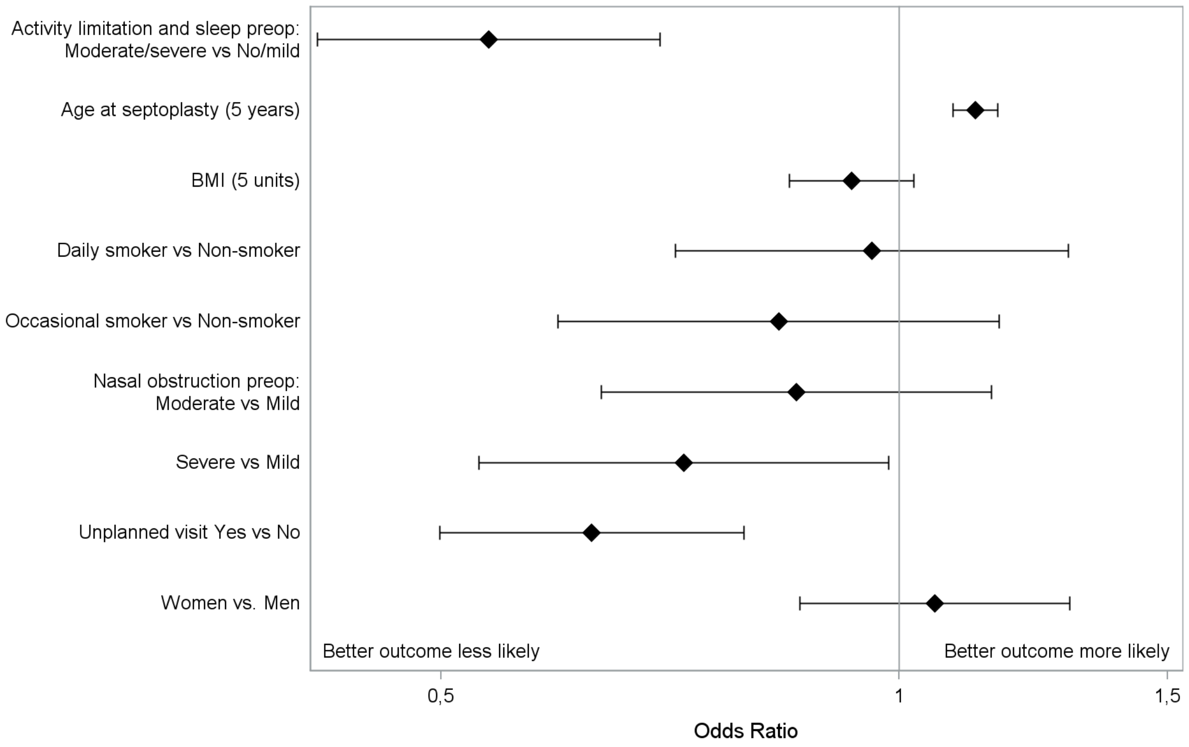

Figure 3. Comparison of nasal obstruction preoperatively between the patients included in the study (answering both the preoperative and postoperative questionnaire, N=2,532) and the patients not included (not answering the postoperative questionnaire, N=2,534). P chi-square=0.01, p trend=0.02

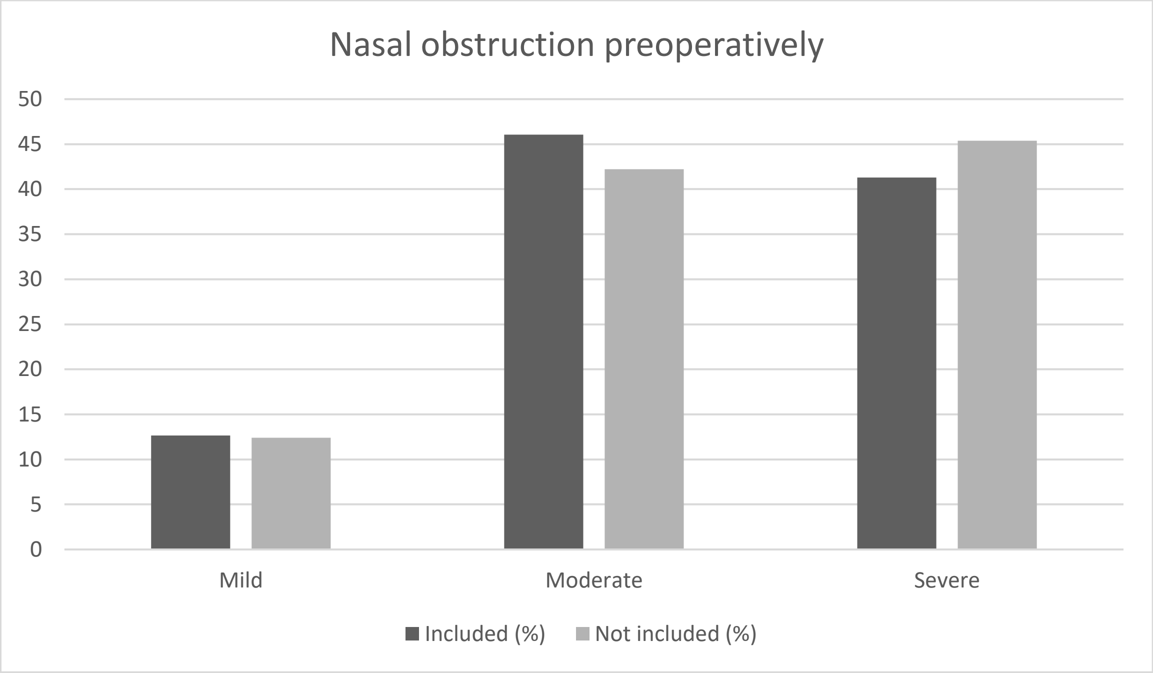

Figure 4. Comparison of nasal obstruction after 12 months between the patients included in the study (answering both the preoperative and postoperative questionnaire, N=2,532) and the patients not included (not answering the preoperative questionnaire, N=675). P chi-square=0.11, p trend=0.03

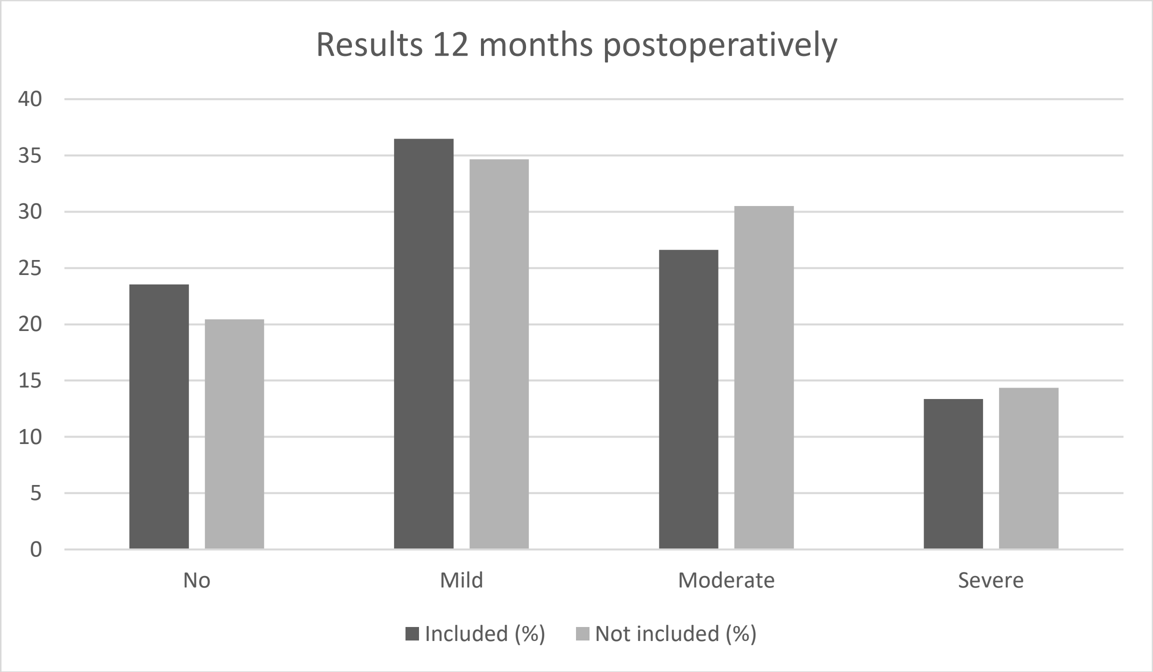

Supplement: Supplementary file 2 [file Datasheet1.pdf]
