# Supplementary material for: A comparison of men and women undergoing septoplasty—the Swedish national septoplasty register
Source: Front Surg. 2023 Jul 31;10:1223607. doi: 10.3389/fsurg.2023.1223607 (PMC10423992; doi:10.3389/fsurg.2023.1223607)
Supplement: Supplementary file 3 [file Datasheet2.pdf]

Septumplastik

Preoperativ enkät A

Gäller endast septumkirurgi med eller utan conchotomi, ej i kombination med annan näs-/bihålekirurgi eller rhinoplastik

IFYLLES AV PATIENTEN

Personnummer:______________________________________

Patientens e-postadress:

Datum för ifyllande av enkäten:______________________

Jag upplever nu
☐ Ingen nästäppa
☐ Mild nästäppa
☐ Måttlig nästäppa
☐ Svår nästäppa

Hur mycket påverkar nästäppan dina dagliga aktiviteter (tex arbete, studier, fritidsaktiviteter) och nattsömn?
☐ Inte alls
☐ Lite grand
☐ Ganska mycket
☐ Väldigt mycket

På vilken sida har du besvär med nästäppa?
☐ Höger
☐ Vänster
☐ Båda sidor

När på dygnet har du besvär?
☐ Dagtid
☐ Nattetid
☐ Såväl dag som nattetid

Röker du?
☐ Ja, dagligen
☐ Ja, ibland
☐ Nej

Längd:____________ (cm) **Vikt:**______________ (kg)

Referensgruppen septumplastikregistret, Cecilia Ahlström Emanuelsson,
ÖNH-kliniken, Skånes universitetssjukhus
Tel: 046-17 10 00
Cecilia.ahlstrom-emanuelsson@skane.se

# Septumplastik<br><br>Preoperativ enkät A	Gäller endast septumkirurgi med eller utan conchotomi, ej i kombination med annan näs-/bihålekirurgi eller rhinoplastik<br><br>**IFYLLES AV LÄKAREN**<br><br>**Personnummer**:______________________________________

Huvuddiagnos

Septumdeviation J34.2	☐ Höger<br>☐ Vänster<br>☐ Bilateralt
**Bidiagnos**	
Rhinit/Allergi(J30, J31)	☐ Ja<br>☐ Nej
Konkahypertrofi(J343)	☐ Ja<br>☐ Nej
Näspolyp(J330)	☐ Ja<br>☐ Nej
Snarkning(R065)	☐ Ja<br>☐ Nej
OSAS (Obstruktiv sömnapné-syndrom), verifierad	☐ Ja<br>☐ Nej
**Rhinometri bedömd som patologisk**	☐ Ja<br>☐ Nej<br>☐ Ej genomfört
**Om ja - vilken mätteknik har använts?**	
Akustisk rhinometri	☐ Ja<br>☐ Nej
Rhinomanometri	☐ Ja<br>☐ Nej
**Tidigare genomgått septumplastik**	☐ Ja<br>☐ Nej
**Planerad operation**	☐Septumplastik utan conchotomi<br>☐Septumplastik med conchotomi

Referensgruppen septumplastikregistret, Cecilia Ahlström Emanuelsson,
ÖNH-kliniken, Skånes universitetssjukhus
Tel: 046-17 10 00
Cecilia.ahlstrom-emanuelsson@skane.se
