# Supplementary material for: A comparison of men and women undergoing septoplasty—the Swedish national septoplasty register
Source: Front Surg. 2023 Jul 31;10:1223607. doi: 10.3389/fsurg.2023.1223607 (PMC10423992; doi:10.3389/fsurg.2023.1223607)
Supplement: Supplementary file 4 [file Datasheet3.pdf]

Septumplastik

Peroperativ enkät B

Personnummer:_______________________________________

Operationsdatum:___________________

Utförd operation ☐Septumplastik utan conchotomi(DJD20)

☐Septumplastik med conchotomi(DJD20+DHB40/45)

Är operatören samma som anmälande läkare? ☐ Ja
☐ Nej

Operationen utförd i narkos? ☐ Ja
☐ Nej

Septumplastik utförd endoskopiskt ☐ Ja
☐ Nej

Operationsteknik

Tunnlar ☐ Unilateralt
☐ Bilateralt

Brosk/ben uttaget ☐ Ja
☐ Nej

Om ja, brosk/ben återinsatt? ☐ Ja
☐ Nej

Plattor ☐ Ja
☐ Nej

Quilting med transseptala suturer ☐ Ja
☐ Nej

Conchotomi kallt stål, shaver ☐ Ja
☐ Nej

Om ja, sida? ☐ Höger
☐ Vänster
☐ Bilateralt

Conchotomi RF, diatermi ☐ Ja
☐ Nej

Om ja, sida? ☐ Höger
☐ Vänster
☐ Bilateralt

Tamponad ☐ Ja
☐ Nej

Om ja, antal dagar? ____________________

Tamponadmaterial

Syntetiskt material absorberande yta, tex Merocel ☐ Ja
☐ Nej

Syntetiskt material med icke absorberande yta, tex Netcell ☐ Ja
☐ Nej

Resorberbar tamponad ☐ Ja
☐ Nej

Gasbindetamponad ☐ Ja
☐ Nej

Andningsrör ☐ Ja
☐ Nej

Annat ☐ Ja
☐ Nej

Om ja, specificera annat material:____________________________

Lokal antibiotika på tamponad ☐ Ja
tex Terracortrildroppar/salva ☐ Nej

Antibiotika

Systemisk antibiotika peroperativt? ☐ Ja
☐ Nej

Systemisk antibiotika postoperativt? ☐ Ja
☐ Nej
