# Supplementary material for: A comparison of men and women undergoing septoplasty—the Swedish national septoplasty register
Source: Front Surg. 2023 Jul 31;10:1223607. doi: 10.3389/fsurg.2023.1223607 (PMC10423992; doi:10.3389/fsurg.2023.1223607)
Supplement: Supplementary file 5 [file Datasheet4.pdf]

Septumplastik

Patientenkät
1 månad postoperativt

Personnummer:____

Du har genomgått en näsoperation för ca 1 månad sedan. För att kunna förbättra vården är det viktigt att få veta om komplikationer har uppstått efter operationen. Vi är angelägna om ditt svar även om allt har varit besvärsfritt.

Datum för ifyllande av enkäten:_____________________

Utöver planerat återbesök, har du besökt sjukvården pga komplikationer till din näsoperation?
☐ Ja
☐ Nej

Om ja, vad var orsaken/orsakerna till besöket?

Blödning ☐ Ja ☐ Nej

Smärta ☐ Ja ☐ Nej

Infektion ☐ Ja ☐ Nej

Annat ☐ Ja ☐ Nej

Om ja, specificera orsak:____

Fick du antibiotika vid detta oplanerade besök? ☐ Ja ☐ Nej

Fick du tillräcklig information inför din operation? ☐ Ja ☐ Nej

12 månader efter genomförd operation kommer du återigen tillfrågas om att besvara en enkät. Om du önskar att den skickas via e-post, ange här aktuell e-postadress:

Tack för din medverkan!

Nationellt Kvalitetsregister Öron-, näs- & halssjukvård

Referensgruppen septumplastikregistret, Cecilia Ahlström Emanuelsson,
ÖNH-kliniken, Skånes universitetssjukhus
Tel: 046-17 10 00
Cecilia.ahlstrom-emanuelsson@skane.se
