# Supplementary material for: A comparison of men and women undergoing septoplasty—the Swedish national septoplasty register
Source: Front Surg. 2023 Jul 31;10:1223607. doi: 10.3389/fsurg.2023.1223607 (PMC10423992; doi:10.3389/fsurg.2023.1223607)
Supplement: Supplementary file 6 [file Datasheet5.pdf]

Septumplastik<br>Patientenkät<br>12 månader post-operativt D

Personnummer:____________________________________

Du har genomgått en näsoperation för ca 12 månader sedan. För att kunna förbättra vården är det viktigt att få veta resultatet och om komplikationer har uppstått efter operationen. Vi är angelägna om ditt svar även om allt har varit besvärsfritt.

Datum för ifyllande av enkäten:____________________

Blev resultatet efter din operation av nässkiljeväggen det du förväntade dig? ☐ Ja ☐ Nej

Om nej, på vilket sätt blev resultatet inte det

förväntade?____

Jag upplever nu
- ☐ Ingen nästäppa
- ☐ Mild nästäppa
- ☐ Måttlig nästäppa
- ☐ Svår nästäppa

Om du fortfarande har besvär med nästäppa:

Hur mycket påverkar nästäppan dina dagliga aktiviteter (tex arbete, studier, fritidsaktiviteter) och nattsömn?
- ☐ Inte alls
- ☐ Lite grand
- ☐ Ganska mycket
- ☐ Väldigt mycket

På vilken sida har du besvär med nästäppa?
- ☐ Höger
- ☐ Vänster
- ☐ Båda sidor

När på dygnet har du besvär?
- ☐ Dagtid
- ☐ Nattetid
- ☐ Såväl dag som nattetid

Har du fått bestående komplikationer efter din operation av nässkiljeväggen? ☐ Ja ☐ Nej

Om ja, vilken/vilka?

☐ Nedsatt luktförmåga	☐ Ja	☐ Nej
☐ Hål i nässkiljeväggen	☐ Ja	☐ Nej
☐ Formförändring av näsan	☐ Ja	☐ Nej
☐ Smärtor i näsan	☐ Ja	☐ Nej
☐ Annat		

Om annat, specificera:_______________________________

Tack för din medverkan!

Referensgruppen septumplastikregistret, Cecilia Ahlström Emanuelsson,
ÖNH-kliniken, Skånes universitetssjukhus
Tel: 046-17 10 00
Cecilia.ahlstrom-emanuelsson@skane.se
